# Identification of high risk anaplastic gliomas by a diagnostic and prognostic signature derived from mRNA expression profiling

**DOI:** 10.18632/oncotarget.5421

**Published:** 2015-09-28

**Authors:** Chuan-Bao Zhang, Ping Zhu, Pei Yang, Jin-Quan Cai, Zhi-Liang Wang, Qing-Bin Li, Zhao-Shi Bao, Wei Zhang, Tao Jiang

**Affiliations:** ^1^ Department of Molecular Neuropathology, Beijing Neurosurgical Institute, Capital Medical University, Beijing, China; ^2^ Department of Neurosurgery, Beijing Tiantan Hospital, Capital Medical University, Beijing, China; ^3^ Center of Brain Tumor, Beijing Institute for Brain Disorders, Beijing, China; ^4^ China National Clinical Research Center for Neurological Diseases, Beijing, China; ^5^ Department of Otolaryngology, Union Hospital, Tongji Medical College, Huazhong University of Science and Technology, Wuhan, China; ^6^ Department of Neurosurgery, The Second Affiliated Hospital of Harbin Medical University, Harbin, China

**Keywords:** anaplastic glioma, mRNA, signature, diagnosis, prognosis

## Abstract

Anaplastic gliomas are characterized by variable clinical and genetic features, but there are few studies focusing on the substratification of anaplastic gliomas. To identify a more objective and applicable classification of anaplastic gliomas, we analyzed whole genome mRNA expression profiling of four independent datasets. Univariate Cox regression, linear risk score formula and receiver operating characteristic (ROC) curve were applied to derive a gene signature with best prognostic performance. The corresponding clinical and molecular information were further analyzed for interpretation of the different prognosis and the independence of the signature. Gene ontology (GO), Gene Set Variation Analysis (GSVA) and Gene Set Enrichment Analysis (GSEA) were performed for functional annotation of the differences. We found a three-gene signature, by applying which, the anaplastic gliomas could be divided into low risk and high risk groups. The two groups showed a high concordance with grade II and grade IV gliomas, respectively. The high risk group was more aggressive and complex. The three-gene signature showed diagnostic and prognostic value in anaplastic gliomas.

## INTRODUCTION

Gliomas are the most common and lethal intracranial tumors [1]. According to the 2007 World Health Organization (WHO) classification [2], they have been divided into different grades. Even within the same WHO grade, the prognosis of patients varied greatly, which revealed the shortcomings of current morphology based classification systems. Thus, great efforts have been spent on finding a more objective and clinically applicable classification of gliomas.

The classification systems based on mRNA expression profiling of glioblastoma multiforme (GBM) or all grades of gliomas have been reported [3–5]. Previous studies classified WHO grade II–IV gliomas into G1, G2 and G2 or high-grade gliomas into proneural, proliferative, and mesenchymal, or GBM into proneural, neural, classical, and mesenchymal molecular subgroups. The predictive [3] and prognostic value [4] of molecular classification of gliomas contribute greatly to personalized medicine. But there are few studies focusing on the classification of anaplastic gliomas alone. Anaplastic gliomas, i.e. WHO grade III gliomas, including anaplastic astrocytoma, anaplastic oligodendroglioma and anaplastic oligoastrocytoma, were reported to have a median overall survival (OS) of 37.6 months [6]. Although it is commonly considered that these tumors often invade neighboring tissue and are able to progress into grade IV secondary glioblastoma multiforme (GBM) [7], there are few studies that could distinguish those tumors from the whole.

In the present study, we obtained whole genome mRNA expression profiling data from Chinese Glioma Genome Atlas (CGGA) as training set and three additional datasets as validation sets. By applying Cox regression, linear risk score formula and receiver operating characteristic (ROC) curve, we pinpointed a three-gene signature. The diagnostic and prognostic value of which were identified and validated in CGGA and validation sets. There were vital differences between low risk and high risk anaplastic gliomas in clinical and molecular features and functional annotations.

## RESULTS

### Prognostic signature identified in CGGA anaplastic gliomas

We previously published mRNA microarray data of 225 Chinese samples (220 WHO grade II–IV gliomas and 5 normal brain controls) [4]. Here, we used 34 anaplastic gliomas from the dataset as training set. Univariate Cox regression analysis adjusted by 10,000 times permutation tests was performed on the 34 anaplastic glioma samples. 1173 probes (1040 genes) were significantly associated with OS (*p* < 0.05, FDR < 0.01). The top 10 prognostic probes were listed in Table 1. To assess the prognostic performance of signatures derived from the top n genes ranked ascendingly by *p* value, we applied ROC curve to obtain a series of AUCs (Supplementary Figure S1). The final signature was derived from the top four probes (three genes), by applying which, we could achieve the maximal AUC (0.9382). The three genes were *GPR85, SHOX2* and *HMBOX1*.

**Table 1:** Top 10 prognostic probes identified by Cox regression

Probe ID	Symbol	Hazard Ratio	β	*P* value	AUC
A_24_P21161	GPR85	0.368	−1.000	1.16E-05	0.7188
A_23_P215687	GPR85	0.135	−2.002	1.55E-05	0.8244
A_24_P300021	SHOX2	3.151	1.148	2.00E-05	0.8846
A_23_P134684	HMBOX1	0.103	−2.273	5.18E-05	0.9382^a^
A_24_P102293	SLITRK5	0.552	−0.594	5.44E-05	0.9182
A_23_P210323	CEP68	0.149	−1.904	5.87E-05	0.9239
A_24_P417526	FRG1B	0.217	−1.528	7.51E-05	0.9346
A_23_P138574	ATE1	0.112	−2.189	7.83E-05	0.9379
A_23_P3602	NUDT7	0.348	−1.056	9.58E-05	0.9290
A_24_P167984	ATMIN	0.052	−2.957	0.0001195	0.9274

a the maximum of AUC.

**Figure 1:**
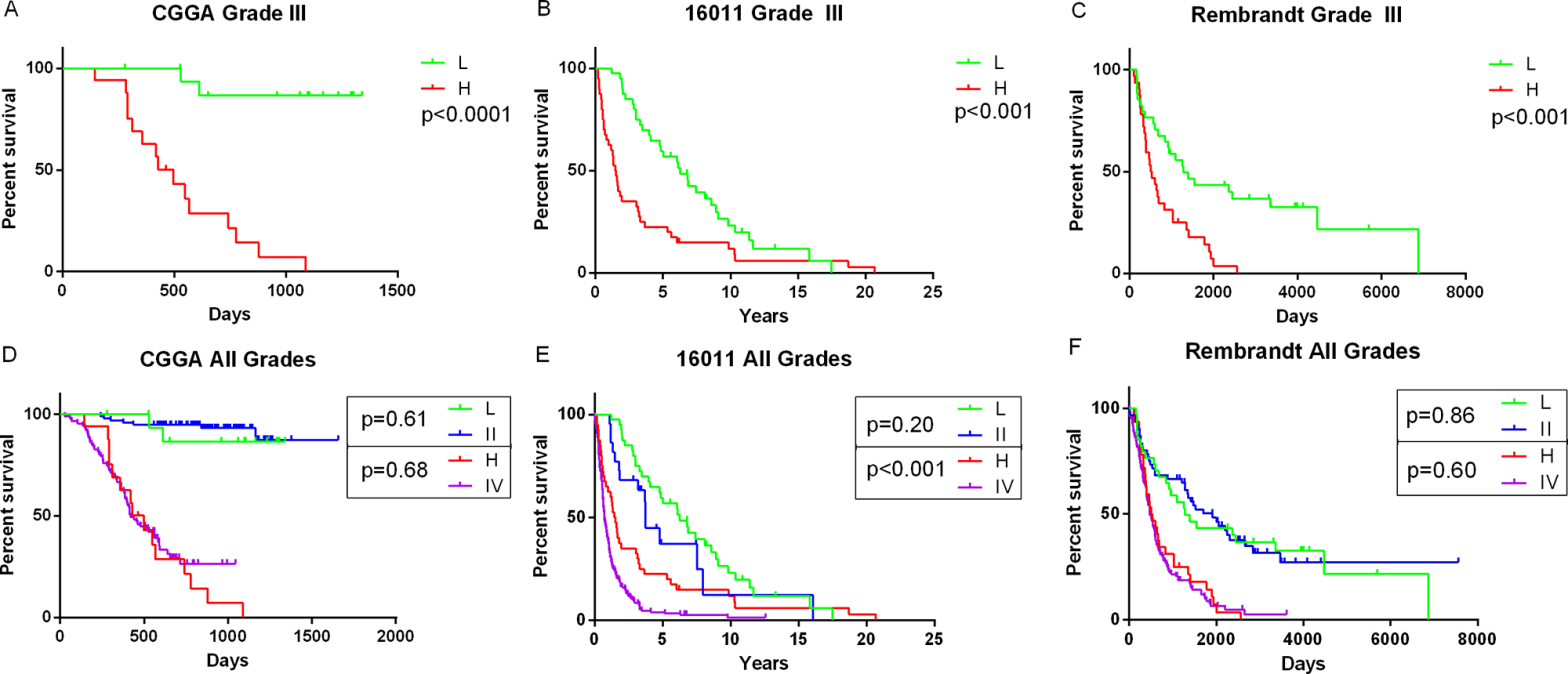
Prognostic value of the signature in training and validation sets and the grade II and grade IV like properties of anaplastic gliomas Patients in low risk group showed a better prognosis than those in high risk group. The two groups also respectively showed similar prognosis with grade II and grade IV gliomas. **A, D.** CGGA data; **B, E.** GSE16011 data; **C, F.** REMBRANDT data; L, low risk group; H, high risk group; II, WHO grade II; IV, WHO grade IV.

**Figure 2:**
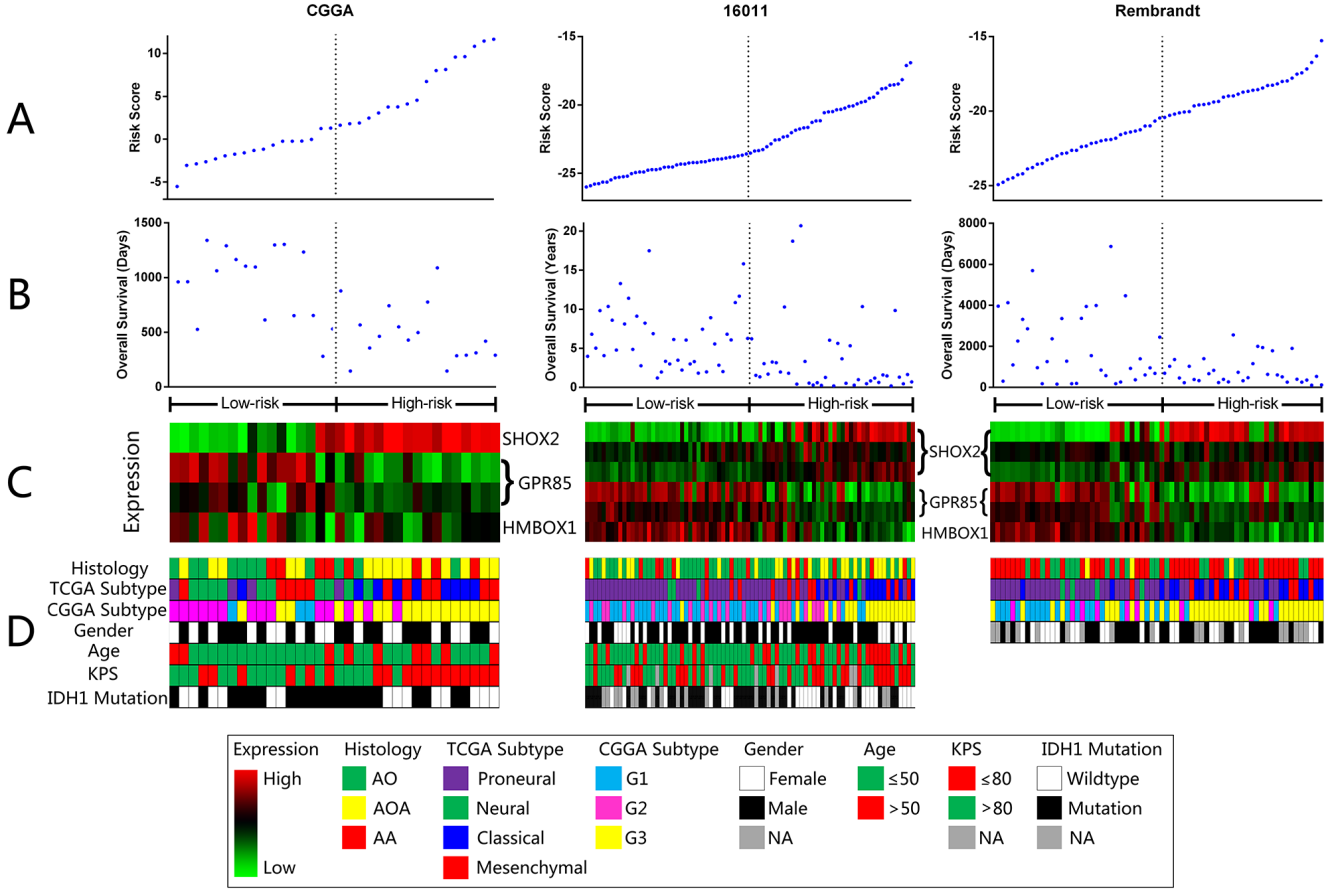
Distribution of risk score, OS, gene expression and clinical or molecular pathological features in CGGA, GSE16011 and REMBRANDT datasets

We then applied the four probes to develop a signature using the risk-score method. The signature risk score was calculated for each of the 34 patients in the training set and then was used to divide them into a high risk group and a low risk group based on the cutoff value (median risk score). We observed that patients in high risk group had a significantly shorter OS than patients in low risk group (*p* < 0.001, Figure 1A). The risk score and OS distribution were shown in Figure 2A and 2B.

### Validation of the prognostic value of the signature in two additional datasets

For the remaining 67, 80 and 263 anaplastic gliomas in REMBRANDT, GSE16011 and TCGA datasets, we used the same β value obtained from the training set to calculate the risk scores. In each validation set, patients were divided into high risk group and low risk group according to the risk score (cutoff: median risk score). The prognostic value of the signatures were validated by all the datasets (*p* < 0.001 for all the three datasets, Figure 1B, 1C and Supplementary Figure S2A) who had results similar to that of the training set. The risk score and OS distribution were also shown in Figure 2A, 2B, Supplementary Figure S2C and S2D.

### The grade II and grade IV like properties of anaplastic gliomas

As was shown in Figure 1D, low risk and high risk anaplastic glioma patients illustrated similar prognosis with grade II (*p* = 0.61) and IV (*p* = 0.68) glioma patients, respectively. Namely, the anaplastic glioma patients displayed distinct grade II and grade IV like properties in prognosis. Similar results were validated in the three validation sets (Figure 1E, 1F, Supplementary Figure S2B).

**Figure 3:**
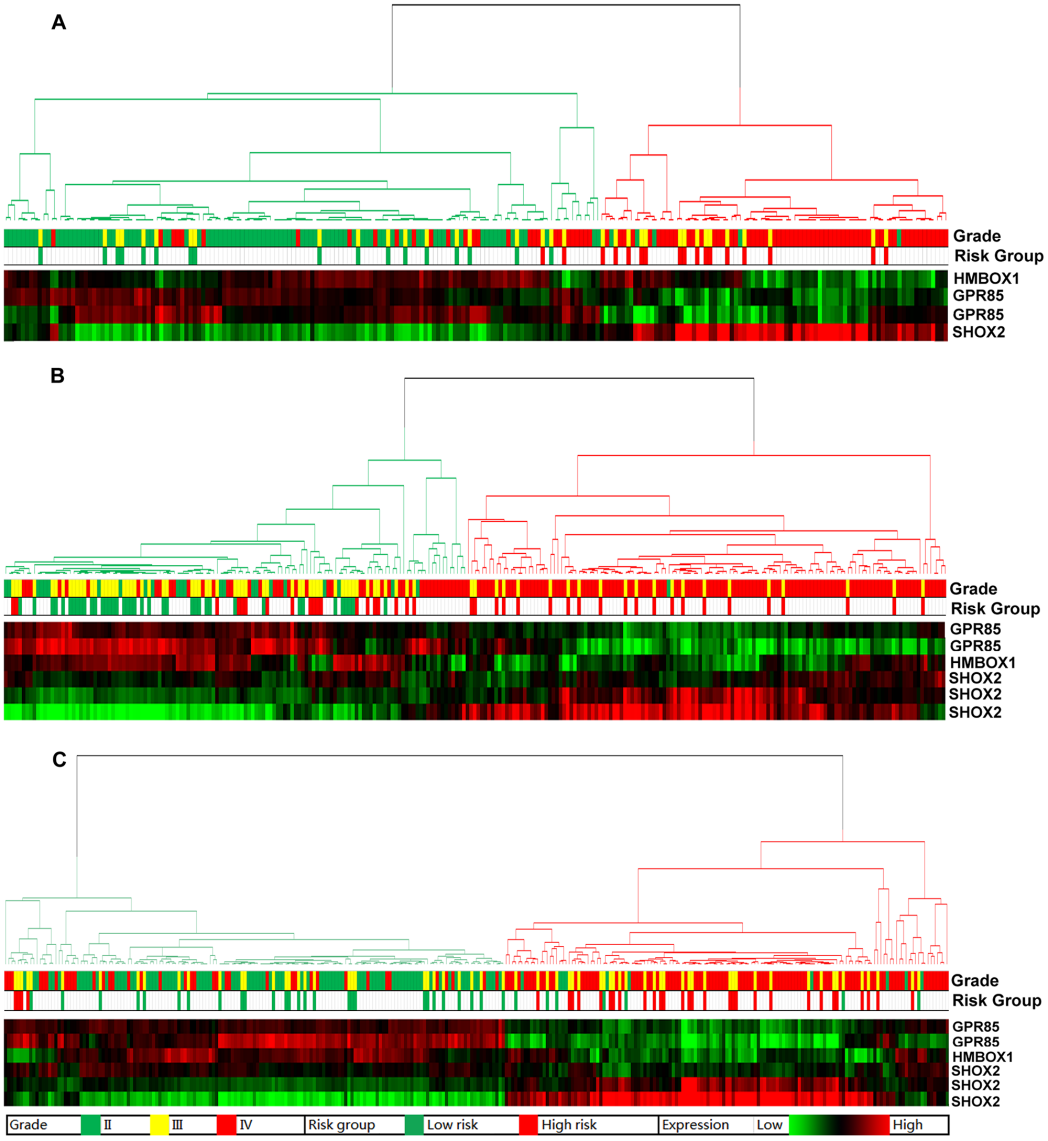
Unsupervised hierarchical clustering of WHO grade II–IV glioma patients based on the expression of the three genes Grade II and grade IV gliomas clustered distinctively while grade III gliomas showed a mix of both branches. The vast majority of low risk anaplastic gliomas (green in risk group) clustered closely to grade II gliomas (green in grade) while the high risk ones (red in risk group) clustered in the branch of grade IV (red in grade). **A.** CGGA data; **B.** GSE16011 data; **C.** REMBRANDT data.

Meanwhile, in order to study the diagnostic value of the signature, we performed hierarchical clustering of all grades of glioma patients in the training set by the expression of the 4 probes. Anaplastic gliomas showed the most variable features compared with the other two grades. The vast majority of low risk anaplastic gliomas clustered closely to grade II gliomas while the high risk ones clustered in the branch of grade IV. The four probes showed definite expression difference between the two branches (Figure 3A). The validation sets showed high consistency with these findings (Figure 3B, 3C and Supplementary Figure S2E). The mutation profile, analyzed in TCGA dataset (Supplementary Figure S2E), also showed similarities to GBM patients: lower IDH1/2, TP53 and ATRX mutation rates and higher EGFR and PTEN mutation rates. The results above suggested that the signature was also a good diagnostic marker for anaplastic gliomas.

### Expression difference of the three genes in low risk and high risk groups

Although the three genes were screened from Cox regression, there was a significant difference in expression between low risk and high risk group. In accordance with previous findings by hierarchical clustering, *SHOX2* was overexpressed in high risk group while *GPR85* and *HMBOX1* showed a reverse situation (Figure 2C and Supplementary Figure S2E). None of the three genes was widely reported in the field of neuro-oncology. *SHOX2*, a member of the homeobox family of genes, encodes a protein characterized as a transcriptional regulator. It has been widely studied in lung cancer as a diagnostic methylation biomarker [8–10]. *GPR85*, also known as *SREB2*, is a members of the G protein-coupled receptor (GPCR) family. It has been found to be a schizophrenia risk factor [11–13]. *HMBOX1* is a transcription factor, associated with differentiation [14, 15] and immune system [16, 17].

### Clinical and molecular features of low and high risk anaplastic glioma patients

To assess the independence of current risk groups with previous widely accepted factors and classification system, we collected and analyzed the corresponding clinical and molecular information of anaplastic glioma patients from the datasets. As was shown in Figure 2D, Supplementary Figure S2E and Table 2, especially in the large dataset of TCGA samples, the risk group showed significant correlation with known prognostic factors (age, histology, IDH gene mutation, pre-operative KPS).

**Table 2:** Characteristics of patients in low risk and high risk group in four datasets

		CGGA		*p*	16011		*p*	REMBRANDT		*p*	TCGA		*p*
		LR	HR		LR	HR		LR	HR		LR	HR	
Sample Size		17	17		40	40		34	33		131	132	
	F	8	8		15	13		12	8		48	65	
Gender	M	9	9	> .05	25	27	> .05	13	16	> .05	83	67	= 0.05
	NA	0	0		0	0		9	9		0	0	
Age		38.4 ± 11.2	45.1 ± 15.3	> .05	43.1 ± 11.8	51.9 ± 13.7	< .01	NA	NA		41.6 ± 12.7	49.3 ± 13.2	< 0.01
	AA	4	4		8	6		20	23		48	76	
Histology	AO	8	3	> .05	25	17	> .05	12	9	> .05	50	32	< 0.01
	AOA	5	10		7	17		2	1		33	24	
	WT	6	8		8	18		NA	NA		9	64	
IDH1 mutation	Mut	11	9	> .05	21	15	> .05	NA	NA		122	68	< 0.01
	NA	0	0		11	7		NA	NA		0	0	
Radiotherapy	Y	17	15		35	31		NA	NA		NA	NA	
	N	0	2	> .05	0	0	> .05	NA	NA		NA	NA	
	NA	0	0		5	9		NA	NA		NA	NA	
Chemotherapy	Y	16	11		9	6		NA	NA		NA	NA	
	N	1	6	> .05	26	27	> .05	NA	NA		NA	NA	
	NA	0	0		5	7		NA	NA		NA	NA	
KPS		87.6 ± 9.2	75.9 ± 12.3	< 0.01	86.2 ± 18.0	80.0 ± 20.8	> .05	NA	NA		NA	NA	

*P* value for age and KPS: *t* test; *p* value for others: chi-square test or Fisher's exact test; LR, low risk group; HR, high risk group; F, female; M male; NA, not available; WT, wild type; Mut, mutation; Y, underwent radiotherapy/chemotherapy; N, not underwent radiotherapy/chemotherapy; KPS, Karnofsky performance status.

In TCGA dataset, we further performed univariate and multivariate cox regression analysis. On univariate analysis, the risk score was significantly associated with survival (*p* < 0.001) along with IDH status, patient age and histology. On multivariate analysis, the risk score was also significant (*p* = 0.036) after adjusting for patient age, histology and IDH gene mutation (Table 3).

**Table 3:** Univariate and mutivariate cox analysis in TCGA anaplastic glioma samples

	Univatiate				Multivariate			
	HR	95% CI		*p*	HR	95% CI		*p*
		Lower	Upper			Lower	Upper	
Gender	0.898	0.549	1.467	0.666				
Age	1.069	1.046	1.092	< 0.001	1.072	1.047	1.097	< 0.001
Histology	1.376	1.039	1.821	0.026	1.390	1.040	1.857	0.026
Risk score	1.146	1.086	1.208	< 0.001	1.069	1.004	1.137	0.036
IDH mutation	0.350	0.211	0.582	< 0.001	0.557	0.288	1.074	0.081

In cox regression analysis: gender was defined as 1, male, 0, female; histology was defined as 1, oligodendroglioma, 2, oligoastrocytoma, 3, astrocytoma; IDH gene mutation was defined as 1, mutated, 0, wild type.

### Functional annotation of the different prognosis

**Figure 4:**
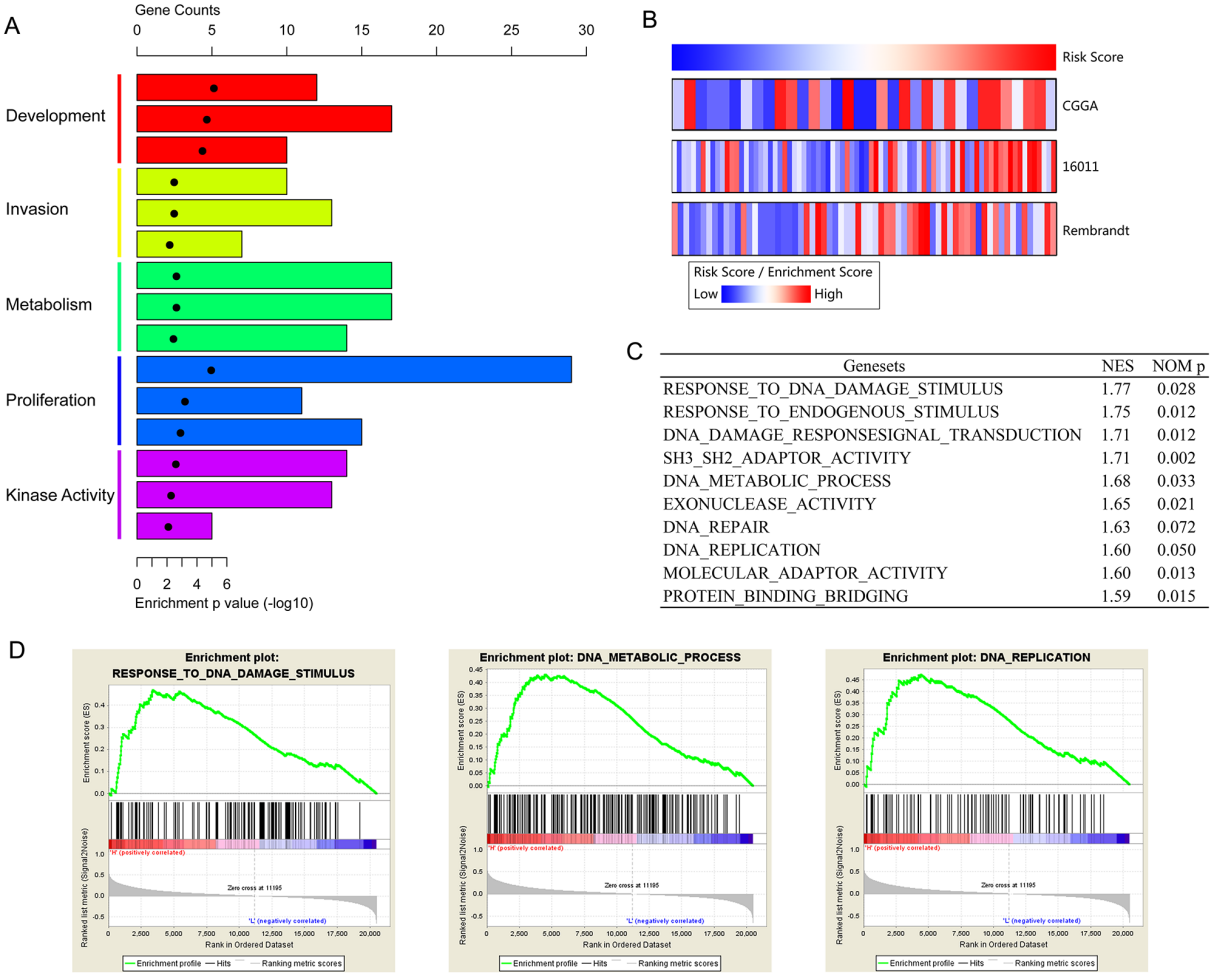
Functional annotation of risk groups **A.** GO analysis revealed the significant association of the genes with increased expression in high risk group with six main pathways. Column height: gene counts; point height: enrichment *p* value. **B.** Gene set variation analysis of proliferation associated genes in three datasets. The risk score (upper panel) was calculated with the formula described above and ranked from left to right. Gene set enrichment score (lower panel) of proliferation was analyzed by GSVA package of R. These genes showed higher expression with the risk score going from low to high. **C.** The top ten enriched pathways in high risk group, analyzed by gene set enrichment analysis of TCGA RNAseq data. **D.** three representative plots of GSEA from C.

In order to find out the functional basis of the notable difference in prognosis, we also performed SAM on low and high risk group in three microarray datasets. After 1000 times of permutation test, those with FDR < 0.1 were considered as differently expressed. And the overlapped genes (401 genes with decreased expression and 308 genes with increased expression in high risk group, Supplementary Table S2) were further analyzed by GO analysis. As was shown in Figure 4A, the oncogenic pathways, such as invasion, proliferation, kinase activity, metabolism, and development were significantly enriched in high risk group. By applying GSVA, the previously reported proliferation associated genes [5] also showed higher enrichment score in high risk group (Figure 4B). We further validated the results in TCGA RNAseq data. GSEA results showed that pathways associated with DNA repair, DNA replication, protein binding, and so on were highly enriched in high risk group (Figure 4C and 4D). It at least partially explained the malignancy and poor survival of patients in high risk group.

## DISCUSSION

The shortcomings of current histopathologic classification, such as a high rate of divergent diagnosis, poor prognostic and predictive value, highlighted the urgent need for an objective molecular based classification. Therefore, much effort have been spent on that of gliomas, which are the most common and lethal intracranial tumors. TCGA, CGGA and many other groups have reported their classification system for GBM alone or all grades of gliomas. But little has been done on anaplastic gliomas. Here we reported a three-gene signature identified and validated by mRNA expression profiling in four independent datasets. Risk score method is a widely used approach to develop a prognostic signature [18–21]. The expression levels of the elements and their independent contribution to prognosis were taken into consideration. By applying the signature, anaplastic gliomas could be divided into low risk and high risk group, which were in high consistency with grade II and grade IV gliomas in molecular and clinical properties. These findings will aid in improving the classification of brain tumors, and the selection of patients with genetically homogeneous tumors for clinical trials.

Anaplastic gliomas were commonly considered to have a medium prognosis between grade II and grade IV. Here, we found that they were more like a group of grade II and grade IV like gliomas based on the expression of three genes: *GPR85, SHOX2* and *HMBOX1*. The two groups showed distinct clinical, molecular features and prognosis, which were extremely similar to that of grade II and grade IV gliomas, respectively. Our findings raised a more practical question: how should patients with anaplastic gliomas be treated? Could a more aggressive therapy benefit the patients in high risk group more? Are we over treating those who are now marked low risk?

Anaplastic gliomas have been usually treated initially by postoperative radiotherapy or chemotherapy alone [22], and the NOA-04 trial had showed similar results between the two schemes [23]. Based on the randomized clinical trials [24, 25], there seemed to be no benefit from radiotherapy following PCV chemotherapy in patients with anaplastic gliomas. But the long-term follow-up of EORTC Brain Tumor Group Study 26951 reported that the addition of six cycles of PCV after 59.4 Gy of RT increases both OS and PFS in anaplastic oligodendroglial tumors [26]. And long-term results of RTOG 9402 also revealed that for patients with 1p/19q codeleted AO/AOA, PCV plus RT may be an especially effective treatment [27].

As there have been reports on subtype specific therapy for anaplastic gliomas or other grades of gliomas, it is reasonable for us to infer that anaplastic gliomas in low risk and high risk group should be given different therapies. A more specific therapy could benefit patients more by giving them a better quality of life.

## MATERIALS AND METHODS

### Datasets and molecular subtype annotation

Whole genome mRNA expression microarray data and corresponding clinical information (histology, gender, age, Karnofsky Performance score (KPS), survival information and isocitrate dehydrogenase 1 (IDH1) gene mutation status) were downloaded from CGGA database (http://www.cgga.org.cn) as training set [4]. The Repository for Molecular Brain Neoplasia Data (REMBRANDT, http://cabig.cancer.gov/solutions/conductresearch/rembrandt/), GSE16011 data (http://www.ncbi.nlm.nih.gov/geo/query/acc.cgi?acc=GSE16011) and The Cancer Genome Atlas (TCGA) RNAseq data (http://cancergenome.nih.gov/) were obtained as validation sets. The RNAseq data were log2 transformed before the following analysis. The characteristics of patients from the four datasets were summarized in Supplementary Table S1. Prediction analysis of microarray (PAM) was performed to annotate the three datasets according to the TCGA and CGGA classification system as previously reported [4].

### Signature development

For preliminary analysis, we first excluded anaplastic samples without survival data or had a overall survival of <60 days. Such short survival times are more likely to be caused by lethal complication rather than gliomas. The signature was developed as previously reported [19–21]. Univariate Cox regression and the corresponding permutation test were performed on the remaining 34 anaplastic glioma patients in CGGA data to get the corresponding Hazard Ratio (HR) and *p* value. After ranking the genes based on *p* value of Cox regression, a list of 1173 probes (1040 genes, *p* < 0.05, FDR < 0.01) were used to developed a linear combination of the gene expression level (expr) weighted by the regression coefficient derived from the univariate Cox regression analysis (β). The risk score for each individual was calculated as follows:

$$\text{Risk score }={\text{expr}}_{\text{gene1}}\times \beta_{\text{gene1}}+{\text{expr}}_{\text{gene2}}\times \beta_{\text{gene2}}+\ldots +{\text{expr}}_{\text{gene n}}\times \beta_{\text{gene n}}.$$

Patients with high risk scores were expected to have poor survival. By applying ROC curve (survivalROC package of R [28], compute time-dependent ROC curve from censored survival data using Kaplan-Meier method), we could keep on adding genes in the list from top to bottom to the signature to get a series of area under the curve (AUC). The final signature was derived from the top four probes (three genes), by applying which, we could achieve the maximal AUC (Supplementary Figure S1). According to the cutoff value (median risk score), patients in the training set were stratified into a high risk group and a low risk group.

### Signature validation

The same β was applied to the validation sets. For the gene *GPR85*, which had two different β values in the training set, we chose the probe A_24_P21161 and the corresponding β value. It had a larger standard deviation (SD), and smaller β value and will be more likely to have a prognostic value with less likely to have a bias. Using SD or median absolute deviation (MAD) to filtering genes with multiple probes are widely used method [3]. And for genes with multiple probes in validation datasets, we used the average expression value of each gene to derive a risk score. For example, for gene A with n probes, the risk score = (β_A_ × probe_1_ + β_A_ × probe_2_ + … + β_A_ × probe_n_)/n. The differences in overall survival (OS) between high risk patients and low risk patients were estimated by using the Kaplan-Meier method and 2-sided log-rank test. The differently expressed genes were identified by significance analysis of microarray (SAM). Those genes with increased expression in high risk patients were used for Gene Ontology (GO) analysis in DAVID (http://david.abcc.ncifcrf.gov/). Gene Set Variation Analysis (GSVA) and Gene Set Enrichment Analysis (GSEA) was also performed for functional annotation [29]. All the statistical analyses were performed by R or GraphPad Prism.

## SUPPLEMENTARY FIGURES AND TABLES




