# Supplementary material for: Identification of high risk anaplastic gliomas by a diagnostic and prognostic signature derived from mRNA expression profiling
Source: Oncotarget. 2015 Sep 28;6(34):36643–51. doi: 10.18632/oncotarget.5421 (PMC4742201; doi:10.18632/oncotarget.5421)
Supplement: Supplementary file 1 [file oncotarget-06-36643-s001.pdf]

SUPPLEMENTARY FIGURES AND TABLES

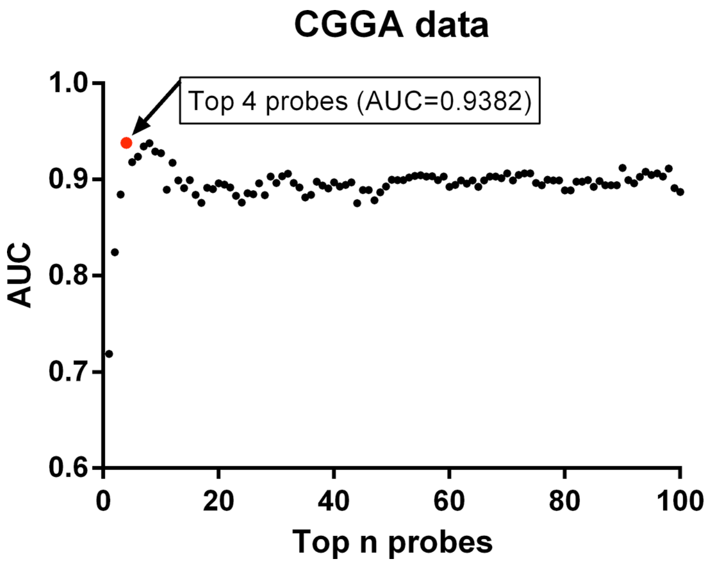

Supplementary Figure S1: A series of AUC derived from linear combination of the top *n* ($1 \leq n \leq 100$) probes in training set (CGGA data). The maximal AUC was developed from the top four probes (three genes), which were further considered as the signature genes in the following analysis.

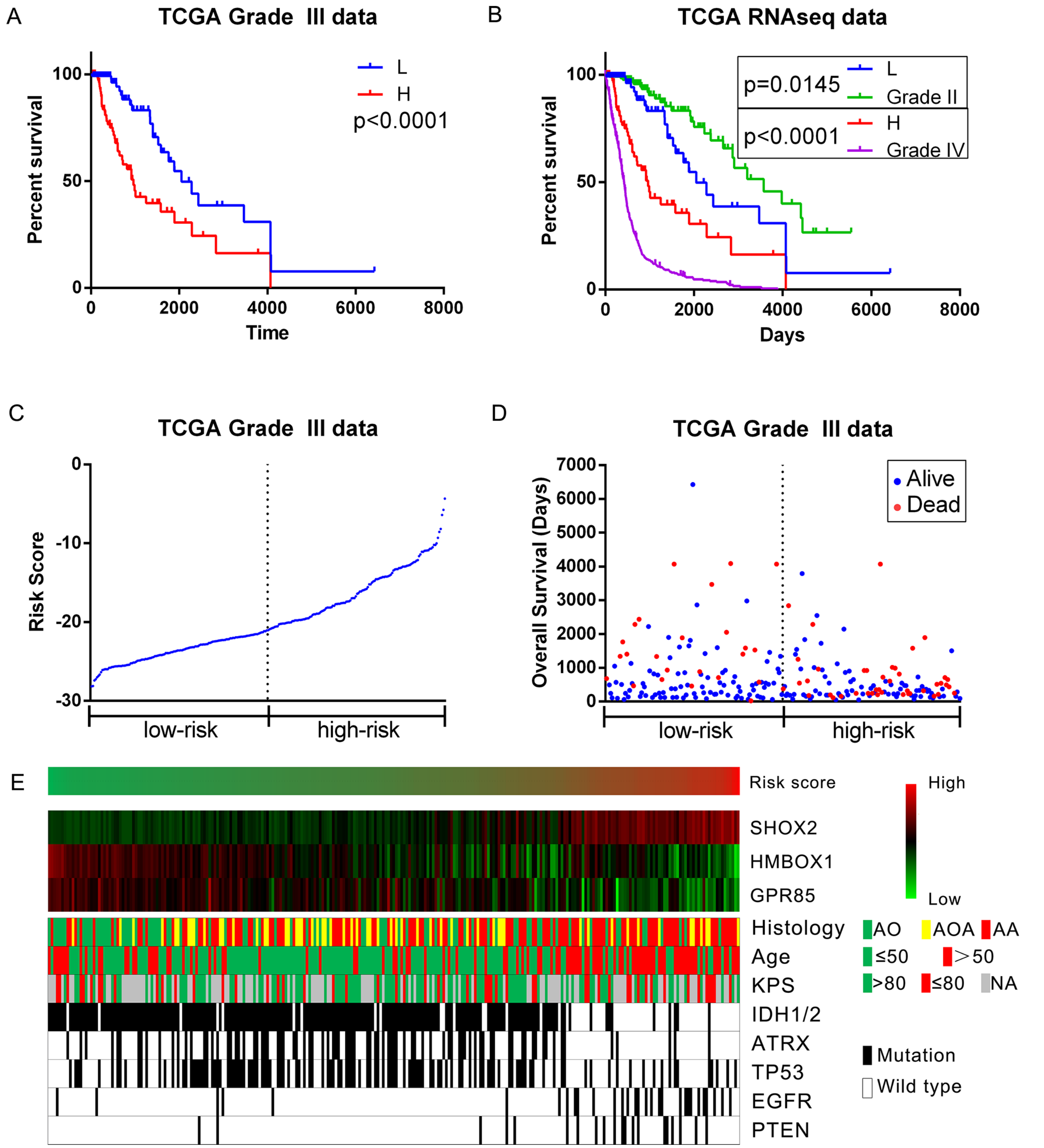

Supplementary Figure S2: Validation results in TCGA RNAseq data. A. Survival analysis revealed that patients in high risk group (H) had a poorer survival than those in low risk group (L) **B.** The high risk group and low risk group were similar to but still different from grade IV and grade II glioma patients, respectively. **C.** The distribution of risk score in TCGA anaplastic glioma samples. **D.** The distribution of overall survival in TCGA anaplastic glioma samples. **E.** The distribution of signature genes expression, clinical and mutation profiles in TCGA anaplastic glioma samples.

Supplementary Table S1: Characteristics of patients in in four datasets

		CGGA			16011			REMBRANDT			TCGA		
**Grade**		**II**	**III**	**IV**	**II**	**III**	**IV**	**II**	**III**	**IV**	**II**	**III**	**IV**
Sample Size		97	34	89	24	85[a]	159	99	84[b]	134	246	263	160[c]
	F	43	16	37	7	30	51	28	25	39	111	113	56
Gender	M	54	18	52	17	55	108	44	39	70	135	150	104
	NA	0	0	0	0	0	0	27	20	25	0	0	0
Age		39.3 ± 9.8	41.7 ± 13.6	45.6 ± 12.8	43.4 ± 12.6	47.6 ± 13.4	54.1 ± 14.0	NA	NA	NA	40.1 ± 13.0	45.6 ± 13.3	59.5 ± 13.5
	A(GBM)	58	8	89	13	16	159	65	58	134	63	124	160
Histology	O	17	11		8	44		30	23		110	82	
	OA	22	15		3	25		4	3		73	57	
	WT	30	14	75	12	29	95	NA	NA	NA	22	73	142
IDH1 mutation	Mut	65	20	14	10	37	33	NA	NA	NA	222	190	9
	NA	2	0	0	2	19	31	NA	NA	NA	2	0	9
KPS		88.8 ± 11.7	81.8 ± 12.2	77.5 ± 11.3	88.7 ± 13.6	80.1 ± 23.4	80.1 ± 17.9	NA	NA	NA	NA	NA	NA

Age and KPS, mean ± SD; F, female; M male; NA, not available; WT, wild type; Mut, mutation; KPS, Karnofsky performance status; a,b, 5 samples from GSE16011 and 17 samples from REMBRANDT were excluded from this study due to lost of follow-up or OS < 60 days; c, in TCGA dataset, only samples with RNAseq data were included in this study.

Supplementary Table S2: The overlapped differently expressed genes between low risk and high risk groups in three microarray based datasets.
